# Microglial pathological synaptic pruning in epilepsy: pathophysiology and therapeutic potential

**DOI:** 10.3389/fimmu.2026.1843165

**Published:** 2026-07-28

**Authors:** Yulei Sun, Donglan Zhou, Jiayi Li, Haonan Wu, Jianmin Liang, Bo Zhang

**Affiliations:** 1 Department of Pediatric Neurology, Children’s Medical Center, The First Hospital of Jilin University, Changchun, Jilin, China; 2 Jilin Provincial Key Laboratory of Pediatric Neurology, Changchun, Jilin, China; 3 Department of Pediatric Respiratory, Children’s Medical Center, The First Hospital of Jilin University, Changchun, Jilin, China

**Keywords:** complement inhibitor, don’t-eat-me signal, eat-me signal, seizures, synaptic plasticity, treatment

## Abstract

Epilepsy affects around 70 million people worldwide, and roughly one-third of them respond poorly to standard antiepileptic drugs. Current treatments are directed almost entirely at neurons, leaving the contribution of non-neuronal cells largely unaddressed. Microglia, the resident immune cells of the central nervous system, both support neuronal survival and shape neural circuits by pruning synapses through “eat-me” and “don’t-eat-me” signals. In epilepsy, this pruning becomes dysregulated: microglia preferentially eliminate inhibitory synapses, producing excitatory/inhibitory (E/I) imbalance, synchronized network discharges, and a lowered seizure threshold. This review summarizes recent work on pathological synaptic pruning by microglia in epilepsy, focusing on the signaling pathways that drive the process and on whether they can be targeted therapeutically in drug-resistant epilepsy. The broader goal is to suggest new treatment directions beyond conventional, neuron-targeted pharmacology.

## 1 Introduction

During the development of the nervous system, neurons form synaptic connections that far exceed functional requirements (1). To establish efficient and functional neural circuits and ensure the proper execution of brain physiological functions, unstable or weak synapses must be selectively eliminated by glial cells, a process known as synaptic pruning (2). As a member of glial cells, microglia play a pivotal role in synaptic pruning. Under physiological conditions, microglia respond to neuronal activity and external stimuli by continuously extending and retracting their processes to monitor synapses (3). In chronic neuroinflammatory environments of various neurological disease models—such as epilepsy, Alzheimer’s disease (AD), multiple sclerosis, and depression—the microglia-mediated synaptic pruning pathways can become hyperactivated in specific brain regions, engulfing normal synapses and neurons. This abnormal phagocytic activity leads to cognitive decline and seizure threshold reduction (4–8).

In patients and animal models of epilepsy, hippocampal microglia exhibit a persistently activated state (9). Activated microglia drive epileptogenesis and exacerbate cognitive deficits via abnormal synaptic pruning at both structural and functional levels. Structurally, three-dimensional serial-section electron microscopy shows that during the formation of spontaneous recurrent seizures (SRS), microglia in the hippocampal cornu ammonis 1 (CA1) stratum radiatum, cornu ammonis 3 (CA3) mossy fiber layer, and dentate gyrus polymorphic layer preferentially engulf glutamate decarboxylase 65 (GAD65)-positive inhibitory presynaptic terminals, while the area of vesicular glutamate transporter 1 (vGluT1)-positive excitatory synapses simultaneously expands. This results in a structural excitatory/inhibitory (E/I) imbalance characterized by a relative increase in excitatory synapses and an absolute reduction in inhibitory synapses (10). Functionally, in direct co-culture models of excitatory neurons and microglia, activated microglia significantly enhance synaptic engulfment, leading to reduced dendritic spine density and a decreased total number of synapses (9). As dendritic spines serve as the structural basis of synaptic plasticity, their loss directly impairs the induction and maintenance of long-term potentiation (LTP), thereby disrupting learning and memory processes. Analyses of epileptic tissue further indicate that the degree of microglial activation positively correlates with seizure duration, frequency, and postoperative cognitive dysfunction (9).

However, the mechanisms by which microglia pathologically prune synapses within epileptic foci remain incompletely defined. Available evidence suggests that epileptic discharges can reprogram the molecular recognition landscape that governs neuron–glia interactions, thereby disturbing the expression level and spatiotemporal distribution of three major classes of signals—find-me, eat-me, and don’t-eat-me cues—and ultimately initiating aberrant synaptic pruning. The following sections therefore examine how these signaling systems are altered in epilepsy, what functions they appear to serve in this context, and which mechanisms are most likely to drive pathological synapse loss. Particular emphasis is placed on their therapeutic implications.

It should be emphasized, however, that the strength of evidence supporting individual mechanisms is highly uneven. The complement component 1q (C1q)–complement component 3 (C3)–complement receptor 3 (CR3) pathway and the γ-aminobutyric acid (GABA)–astrocyte C3–complement C3a receptor (C3aR) cascade currently represent the best-substantiated mechanisms, having been supported by findings from both rodent epilepsy models and resected human tissue (11–14). By contrast, the proposed involvement of triggering receptor expressed on myeloid cells 2 (TREM2) (4, 15), apolipoprotein E (ApoE) (16–18), CUB and Sushi multiple domains 1 (CSMD1) (19, 20), and myeloid differentiation factor 2–C-type lectin domain containing 7A (MD2–CLEC7A) (21, 22) in epileptic synaptic pruning remains far more tentative. In most cases, the rationale for implicating these pathways is extrapolated from AD, ischemic stroke, tauopathy, or other neurological disorders, whereas direct epilepsy-specific evidence is still limited and largely confined to genetic association or indirect mechanistic inference. Accordingly, throughout this Review, we aim to distinguish as clearly as possible between mechanisms for which a more direct causal role has been established in epilepsy models and those that remain speculative extensions from related disease contexts.

## 2 Find-me signals

**Figure 1:**
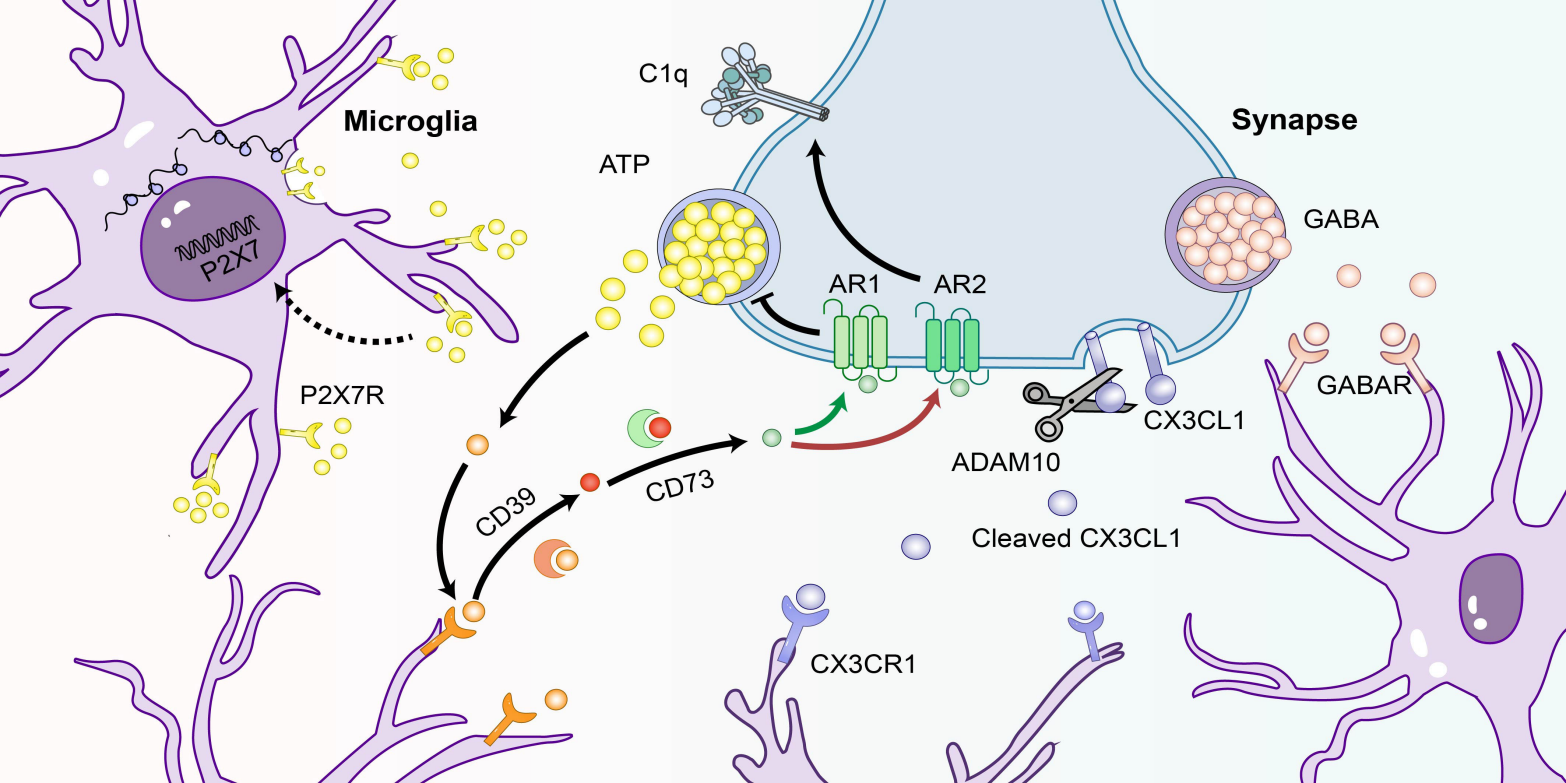
Find-me signals recruiting microglia to vulnerable synapses in epilepsy. Schematic illustration of how neuronal hyperactivity in epilepsy mechanistically engages microglial chemotactic machinery through convergent purinergic, fractalkine, and GABAergic pathways. Seizure-driven neuronal depolarization triggers activity-dependent release of ATP into the extracellular space, where it operates through two mechanistically distinct arms. In the direct arm, ATP binds microglial P2X7 receptors (P2X7R); sustained P2X7R activation gates Ca^2+^ influx, drives cytoskeletal remodeling, and promotes directed process extension toward the active synapse, while simultaneously upregulating P2X7R transcription to amplify microglial responsiveness (dashed arrow). In the indirect arm, extracellular ATP is sequentially hydrolyzed by ectoenzymes CD39 (ATP→AMP) and CD73 (AMP→adenosine); the resulting adenosine engages presynaptic A1R (Gi-coupled, suppressing further ATP release through negative feedback) and A2AR (Gs-coupled, modulating synaptic vesicle dynamics), thereby fine-tuning the spatiotemporal gradient of the find-me cue. In parallel, the membrane-tethered chemokine CX3CL1 is proteolytically cleaved by the metalloproteinase ADAM10—whose activity is itself elevated under epileptic conditions—releasing soluble CX3CL1 that establishes a long-range chemotactic gradient sensed by microglial CX3CR1. GABA spillover from hyperactive inhibitory terminals additionally engages microglial GABA receptors, linking inhibitory neurotransmission directly to microglial surveillance state. Together, these convergent signals (i) localize microglial processes to specific synapses, (ii) prime microglia for subsequent recognition of eat-me cues, and (iii) initiate C1q deposition that bridges find-me to pruning execution.

Following seizures, neurons and microglia undergo profound pathophysiological changes, including membrane potential dysregulation, ionic imbalance, and cytoskeletal disruption. These changes trigger cascades of molecular signaling. As an active recruitment mechanism, injured neurons release a repertoire of soluble ‘find-me signals’, including the purinergic transmitter adenosine triphosphate (ATP), the chemokine C-X3-C motif chemokine ligand 1 (CX3CL1; fractalkine), and the inhibitory neurotransmitter GABA. These signals orchestrate microglial migration and activation, initiating the clearance program. Notably, these signals exhibit both quantitative diversity and high spatiotemporal specificity, providing guidance and localization for the precise exposure of subsequent “eat-me” signals. The mechanism of this part is illustrated in **Figure 1**.

### 2.1 ATP

ATP acts both as a chemotactic signal and as a metabolic substrate, and different purinergic receptors determine whether microglia respond with protective migration or with pathological pruning. Under physiological conditions, ATP–P2Y12 receptor (P2Y12R) signaling is required for maintaining normal microglial morphology and surveillance. This pathway supports baseline microglial morphology, process motility, and branching complexity. When P2Y12R is lost, microglia develop more numerous but shorter, less spherical branches, become less efficient at surveillance, and contact neuronal somata more often (23).

In epilepsy, extracellular ATP rises rapidly. At the early stage, as a chemoattractant, ATP binds to P2Y12R, triggering directional extension of microglial pseudopodia within seconds. This response quickly recruits microglia to hyperexcitable brain regions (24–26). ATP is hydrolyzed into adenosine (ADO) via the ectonucleoside triphosphate diphosphohydrolase 1 (CD39)/ecto-5’-nucleotidase (CD73) ectoenzyme cascade. ADO then produces different effects through different receptors. ADO–adenosine A1 receptor (A1R) signaling suppresses neuronal firing and shows anti-epileptic effects. In contrast, excessive ADO–adenosine A2A receptor (A2AR) signaling depolarizes cell membranes, enhances synaptic excitotoxicity, and increases C1q expression. This process promotes microglia-mediated abnormal pruning of glutamatergic synapses and accelerates degeneration of hippocampal circuits (27–31).

During status epilepticus (SE), extracellular ATP can increase to ≥0.3 mM, which activates the low-affinity P2X7 receptor (P2X7R). P2X7R activity attenuates miR-22–mediated translational inhibition of *P2RX7* mRNA, thereby upregulating receptor expression. The P2X7R–NLR family pyrin domain containing 3 (NLRP3)–interleukin-1 beta (IL-1β) signaling axis subsequently activates microglia and astrocytes, exacerbating inflammation and disrupting synaptic plasticity. Consequently, the epileptic brain may enter a vicious cycle of hyperexcitation, ATP release, inflammation, and excessive synaptic pruning (32–34). Clinical evidence also supports this shift in receptor balance. In patients with severe hippocampal sclerosis and drug-resistant temporal lobe epilepsy (TLE), cortical P2Y12R expression is markedly reduced, whereas P2X7R expression is significantly increased (23, 35).

Beyond its pro-inflammatory role, P2X7R signaling has recently been connected to the transcriptional control of microglial inhibitory synapse phagocytosis. Seizure-induced neuronal ATP release activates microglial P2X7R, leading to calcium influx and phosphorylation of calcium/calmodulin-dependent protein kinase II (CaMKII), which promotes nuclear export of histone deacetylase 5 (HDAC5) and de-repression of the transcription factor myocyte enhancer factor 2A (MEF2A) (36). MEF2A then binds the promoters of differentiation 74 (CD74) and NIMA-related kinase 7 (NEK7) to coordinately enhance NLRP3 inflammasome assembly and microglial phagocytic activity, as confirmed by chromatin immunoprecipitation and luciferase reporter assays (36). MEF2A immunoreactivity is elevated in activated amoeboid microglia in human refractory TLE resection tissue relative to trauma controls, and in the kainic acid (KA) mouse model peaks at 3 days post-injury before partially declining by day 7, paralleling the acute-phase course of microglial activation (36). Functionally, microglia-specific MEF2A knockdown or pharmacological inhibition reduces microglial engulfment of GABA type A receptors (GABA_A_Rs), decreases C3 deposition at gephyrin-positive postsynaptic sites, restores inhibitory receptor expression (*Gabra1, Gabra3, Gabrb2, Gabrg2*), and lowers seizure frequency, whereas excitatory receptor–associated proteins remain unchanged (36). Notably, both MEF2A inhibition and the GABA-driven mechanism described by Chen et al. (28) are accompanied by reduced C3 deposition at gephyrin-positive inhibitory synapses, suggesting that distinct upstream signals—ATP–P2X7R acting on microglia and GABA acting on astrocytes—may ultimately converge on complement-tagged inhibitory synapse elimination, although whether the MEF2A pathway directly engages astrocytic C3 production remains to be determined.

In epileptic networks, ATP therefore plays two roles: chemoattractant for microglial migration and, through ADO/P2X7R pathways, regulator of synaptic engulfment. Its metabolic fate and receptor environment together influence whether synapses are preserved or eliminated, and both P2Y12R and P2X7R have shown therapeutic potential in animal models (discussed further in the treatment section).

### 2.2 CX3CL1

The CX3CL1–C-X3-C motif chemokine receptor 1 (CX3CR1) axis contributes to microglial chemotaxis and synaptic pruning in epilepsy. In normal development, membrane-bound CX3CL1 in the barrel cortex and hippocampus is cleaved by a disintegrin and metalloproteinase 10 (ADAM10) to generate soluble fractalkine, which engages CX3CR1 to guide moderate synaptic pruning and match synapse maturation to sensory input. CX3CR1 deficiency reduces microglial recruitment and pruning, transiently weakening baseline synaptic transmission and connectivity (37, 38). In epilepsy, neuronal CX3CL1 and microglial CX3CR1 are both upregulated, which may amplify chemoattractant signaling and concentrate CX3CR1-positive microglia in hyperexcitable regions (39). Enhanced recruitment of this kind has been linked to excess synaptic engulfment, microglial activation, and postictal neuronal injury, although whether CX3CR1-driven pruning preferentially affects inhibitory or excitatory synapses in epilepsy has not been tested directly (39). Most of the available evidence comes from developmental pruning studies (37, 38) and a small number of seizure models (39), and direct quantification of synapse-type selectivity and spontaneous seizure outcomes following CX3CR1 manipulation in epilepsy remains lacking. CX3CL1–CX3CR1 therefore looks like a homeostatic chemotactic axis that can be redirected toward pathological recruitment in epilepsy. It is a plausible target, but the case needs more direct mechanistic work.

### 2.3 GABA

Beyond its canonical role as an inhibitory neurotransmitter, GABA may also function as a context-dependent find-me signal that helps recruit microglia toward inhibitory synapses. During cortical development, microglia express GABA receptors and detect GABA released by inhibitory neurons, eliciting a chemotactic response broadly analogous to that induced by ATP and thereby contributing to the refinement of inhibitory circuits (11, 40, 41) modeling. *In vivo* imaging further shows that GABAergic axons generate numerous transient synaptic protrusions during normal development, only a subset of which are ultimately stabilized and matured in a GABA-dependent manner (42). Conditional deletion of GAD65 or the vesicular GABA transporter (vGAT) in inhibitory interneurons stabilizes presynaptic boutons, increases synaptic density, and impairs axonal branch retraction (42). GABAergic signaling, in other words, is not merely permissive for inhibitory transmission but participates directly in the activity-dependent selection and remodeling of inhibitory synapses.

In epilepsy, this developmental signaling axis appears to be redeployed in a maladaptive manner. In the TLE microenvironment, hyperactive inhibitory interneurons release excess GABA, which engages the GABBR2 subunit of the microglial GABA type B receptor (GABA_B_R; a functional heterodimer composed of GABBR1 and GABBR2 subunits) and directs their processes toward GABAergic terminals, initiating selective pruning of inhibitory synapses while excitatory synapses are relatively spared, thereby promoting network hyperexcitability (11). This GABA-directed recruitment does not act in isolation but operates upstream of the astrocytic complement response detailed in a later section, in which GABA simultaneously activates astrocytic signaling to generate C3 that tags inhibitory synapses for microglial engulfment (11). The evidence for GABA as a find-me signal in epilepsy comes essentially from one mechanistic study, validated in both a kainic acid mouse model and human TLE tissue (11). How much of the inhibitory-synapse loss is driven by direct microglial GABA sensing rather than by astrocyte-mediated complement tagging has not been fully resolved. Even so, GABA looks like a signal that shifts from guiding physiological refinement of inhibitory circuits toward driving pathological inhibitory-synapse loss in epilepsy, and that shift is a plausible target for intervention.

## 3 Eat-me signals

**Figure 2:**
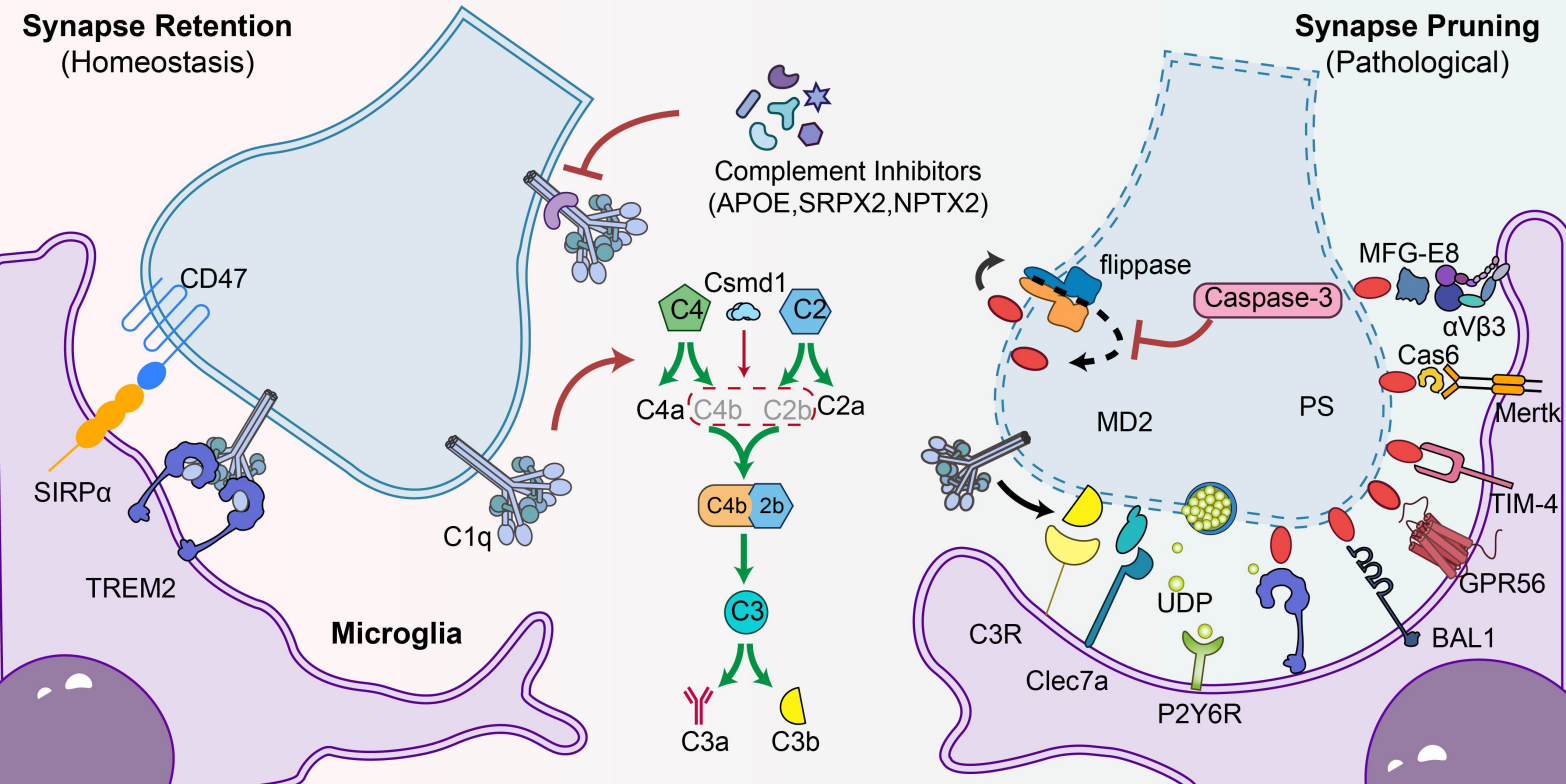
Molecular checkpoints determining synapse retention versus pathological pruning. Mechanistic schematic of the eat-me/don’t-eat-me balance that gates microglial phagocytosis of synapses in epilepsy. Under homeostatic conditions (left), multiple layered safeguards actively protect synapses: (i) synaptic CD47 engages microglial SIRPα, recruiting SHP-1/SHP-2 phosphatases that dephosphorylate phagocytic signaling intermediates and thereby suppress engulfment “in cis”; (ii) secreted complement regulators (APOE, SRPX2, NPTX2) bind and sequester C1q, preventing its docking on synaptic surfaces; (iii) Csmd1 accelerates decay of nascent C3 convertase, aborting downstream amplification; (iv) TREM2 supports microglial homeostatic surveillance without triggering full phagocytic commitment. The net effect is that even when C1q transiently contacts synapses, opsonization is restrained and pruning is not licensed. Under epileptic conditions (right), this balance collapses through several converging mechanisms. First, the classical complement cascade is unleashed (center): C1q binds aberrant synaptic surfaces and activates C4 and C2, generating the C4b2b convertase that cleaves C3 into C3a (anaphylatoxin) and C3b (opsonin); deposited C3b tags synapses for recognition by microglial CR3/C3R, while diffusible C3a engages C3aR to amplify microglial recruitment. Second, caspase-3 activation in stressed synapses inhibits the aminophospholipid flippase, abolishing inward translocation of phosphatidylserine (PS) and resulting in PS externalization on the synaptic outer leaflet. Externalized PS is recognized through redundant routes: directly by TIM-4, GPR56, and BAI1; and indirectly via the bridging molecules MFG-E8 (linking PS to microglial integrin αVβ3) and Gas6 (linking PS to Mertk). Third, hydrolysis of synaptic UTP generates UDP, which engages microglial P2Y6R and licenses phagocytic cup formation, while Clec7a recognizes carbohydrate “altered-self” patterns on damaged synapses. The simultaneous loss of don’t-eat-me protection and gain of multiple, partially redundant eat-me cues lowers the threshold for engulfment and explains why pruning becomes both excessive and selective in the epileptic brain.

The dysregulated find-me signals described above initiate microglial migration toward vulnerable synapses, representing the first step of pathological synaptic pruning. The subsequent recognition and engulfment of synapses depend on eat-me signals, which become enriched at synaptic membranes under conditions of neuronal hyperexcitation, metabolic stress, or injury. These signals include components of the complement cascade, such as C1q and C3 cleavage fragments, as well as externalized phosphatidylserine (PS) on the synaptic membrane. The exposure or accumulation of these signals increases the likelihood that affected synapses are recognized and removed by microglia, although the molecular determinants governing which synapses are tagged remain incompletely defined. These eat-me signaling pathways are detailed in the following sections and illustrated in **Figure 2**.

### 3.1 Complement and complement inhibitors

#### 3.1.1 Complement

The complement system, originally defined as a component of innate immunity, also functions as an eat-me signaling pathway in microglial synaptic pruning, both in development and in disease reviewed in (43). Activation proceeds through three convergent recognition pathways, classical, alternative, and lectin, all culminating in C3 cleavage, C5 convertase formation, and assembly of the terminal membrane attack complex (MAC; C5b-9). During development, classical-cascade pruning refines synaptic connectivity in the retina-lateral geniculate pathway (44), visual thalamus (45), and sensorimotor cortex (46): C1q and C3 cleavage products, especially inactivated complement component 3b (iC3b), preferentially accumulate on less active synapses, where they facilitate CR3-dependent microglial engulfment in an activity-dependent manner.

In epilepsy, pathological complement activation has been documented in multiple human subtypes and experimental models. Aronica et al. provided the earliest systematic evidence, showing that C1q, C3, and complement component 4 (C4) mRNA and protein were elevated in both a rat TLE model and in surgically resected human TLE hippocampi with hippocampal sclerosis. C1q and C3d immunoreactivity appeared in astroglia, microglia, and neurons—particularly in regions of cell loss—and terminal activation products were mostly associated with activated microglia (47). Wyatt et al. then confirmed elevated C1q and iC3b at the protein level in human drug-resistant TLE resections, with C1q co-localizing with microglia and dendrites and with phagocytic signaling molecules upregulated (13). The same pattern extends beyond TLE: C3 is elevated in tuberous sclerosis complex (TSC) and focal cortical dysplasia (FCD) type IIb lesions, where complement factors co-localize with astroglia, microglia, neurons, and the abnormal balloon and giant cells, making complement overactivation a shared feature of these mechanistic target of rapamycin (mTOR)–related disorders (mTORopathies) (48). In rodent models, a single episode of SE triggers hippocampal C1q and C3 signaling that persists from days to months and correlates with spontaneous seizure frequency in chronic TLE (49). C3 also remains elevated at the chronic stage of pilocarpine-induced TLE, with levels tracking seizure severity and hippocampal-dependent cognitive deficits (50). The temporal pattern is consistent: complement activation in epilepsy persists from acute injury through chronic disease.

Genetic and pharmacological work has begun to pin down how C1q contributes to epileptic synapse loss. One proposed mechanism is that C1q recognizes phosphatidylserine-like eat-me signals on stressed synaptic membranes (51), with local non-apoptotic caspase-3 activation at synapses facilitating C1q-mediated tagging (52). After febrile seizures, caspase-3 activation enables C1q tagging of inhibitory presynapses, leading to CR3-dependent microglial engulfment, reduced inhibitory synapse density, and increased circuit excitability (12). Genetic deletion of C1q, however, produces the opposite phenotype: a failure to prune excess neocortical excitatory synapses, enhanced glutamatergic connectivity, and spontaneous behavioral seizures with concurrent bihemispheric spike-and-slow-wave electroencephalographic (EEG) activity (2, 53). Appropriate C1q-mediated excitatory pruning therefore appears important for normal circuit stability. The two opposing phenotypes, too little pruning when C1q is absent, too much when it is overactivated in acquired epilepsy—mean that complement’s role in seizure threshold is context-dependent, and that a useful therapy would need to suppress pathological complement activation without disabling the cascade’s normal functions. In mild traumatic brain injury, for example, chronic C1q deposition on thalamic GABAergic synapses drives microglial selective pruning and produces epileptiform activity that anti-C1q antibodies can reverse (14), a mechanism distinct from, but potentially complementary to, the GABA–astrocytic C3 axis described above.

Downstream of C1q, C3 cleavage produces complement component 3b (C3b) and iC3b, which coat synaptic membranes as direct ligands for microglial CR3-dependent engulfment. Mice lacking C3 are protected against recognition memory deficits and reactive astrogliosis after pilocarpine-induced SE, although initial seizure severity is unaffected (54). This places C3 signaling on the cognitive-comorbidity side of epilepsy rather than on seizure threshold itself, and identifies it as a disease-modifying molecule. The C3–C3aR axis is not exclusively driven by classical complement initiation, however. Under some pathological conditions, C3 is deposited at synapses through the alternative pathway without preceding C1q elevation. In demyelinating disease models, synapse loss correlates with elevated synaptic C3 without significant C1q upregulation (8), and in perioperative neurocognitive disorder, C3-dependent excitatory synapse engulfment proceeds through a C1q-independent route (55). The implication for epilepsy is that complement-mediated pruning probably involves both classical C1q-initiated and C1q-independent C3 activation, so anti-C1q neutralization, useful as it is in certain acquired epilepsy contexts (14, 56), may not fully attenuate complement-driven synapse loss if the alternative pathway is concurrently upregulated.

The terminal complement pathway provides a second effector arm. C5 convertases cleave complement component 5 (C5) into C5a and C5b; C5b initiates MAC assembly, and direct intracerebral injection of MAC into rodents evokes seizures and neurodegeneration (57). Terminal complement activation is therefore not just a downstream readout, it can actively contribute to network hyperexcitability. C5a, the soluble anaphylatoxin released by this cleavage, binds C5a receptor 1 (C5aR1) on microglia and triggers pro-inflammatory signaling, including tumor necrosis factor α (TNFα) release and changes in microglial K^+^ outward currents; C5aR1 is upregulated in the hippocampus in both the pilocarpine and intrahippocampal KA models after SE (58). C5a–C5aR1 signaling looks like a distinct node in complement-mediated pruning, separable from C3aR-dependent engulfment. Gomez-Arboledas et al. showed in two AD mouse models that C1q tagging of synapses is not on its own sufficient to trigger microglial ingestion of excitatory terminals—C5aR1 signaling is required as a second step, and genetic ablation or pharmacological inhibition of C5aR1 partially rescues excessive presynaptic pruning and synapse loss in an age- and region-dependent manner (59). This synapse-level evidence comes from AD rather than epilepsy, but it raises the possibility that downstream C5a–C5aR1 signaling gates the transition from complement opsonization to active engulfment. The idea is testable in epilepsy, where C1q and C3 are concurrently upregulated (13, 47) and yet not all complement-tagged synapses are eliminated equally (10).

Of all the eat-me mechanisms reviewed here, the C1q–C3–CR3 axis is the best characterized in epileptic synaptic pathology. The supporting evidence is broad: human resection tissue across TLE, TSC, and FCD (13, 47, 48); SE-induced rodent models (49, 50); genetic rescue experiments (53, 54); and pharmacological intervention (14, 56). Overactivation of this axis drives pathological microglia-mediated synapse elimination and is among the most strongly supported mechanisms linking complement to both epileptogenesis and the associated cognitive impairment. Because the cascade is context-dependent and altered pruning can affect network excitability in either direction, the practical question for therapy is how to suppress pathological complement activation without disabling its physiological functions. That balance has not yet been systematically tested in epilepsy-specific preclinical models.

#### 3.1.2 Complement inhibitors

As described above, overactivation of the complement cascade, particularly the deposition of C1q and C3 cleavage fragments at synaptic membranes, is one of the best-supported mechanisms driving microglia-mediated pathological synaptic pruning in epilepsy, and if left unchecked may contribute to excessive synapse loss. Under physiological conditions, this process is constrained by a set of endogenous complement regulators expressed in the nervous system, which limit the intensity of complement-dependent eat-me signaling by inhibiting activation of the classical and alternative pathways at distinct steps of the cascade. These regulators help maintain a balance between restraining excessive synapse elimination and preserving the protective functions of complement-mediated immune surveillance. Several such regulators—including neuronal pentraxins, CSMD1, ApoE, and sushi repeat-containing protein X-linked 2 (SRPX2)—have been implicated in epilepsy through genetic association, expression changes, or functional studies in related disorders, although direct evidence that their dysfunction drives pathological pruning in epilepsy remains limited. Restoring or augmenting the activity of these endogenous brakes represents a conceptually distinct therapeutic strategy that targets the regulatory arm of the complement system rather than its effector components.

##### 3.1.2.1 NPTX2

Neuronal pentraxin 1 (NPTX1; also known as NP1) and neuronal pentraxin 2 (NPTX2; also known as NP2 or neuronal activity-regulated pentraxin, NARP) belong to the neuronal pentraxin family, which organizes excitatory and inhibitory synapses in part through the presynaptic neuronal pentraxin receptor (54). Beyond their structural role, neuronal pentraxins have been reported to modulate complement-dependent synaptic pruning, but their reported effects differ in direction across contexts. On one hand, neuronal pentraxins can co-localize with and bind C1q at synapses; NPTX1 has been implicated in promoting C1q-associated synaptic pruning in adult mice (60). On the other hand, in neurodegenerative models NPTX2 restrains microglia-mediated synapse loss by inhibiting complement activity, and NPTX2 deletion leads to excessive microglial pruning of excitatory synapses (61), indicating a complement-inhibitory function for NPTX2 that is opposite in direction to the C1q-promoting activity attributed to NPTX1.

These context-dependent activities are also evident across the temporal stages of epilepsy. In acute seizure models, NPTX2 expression is upregulated and enhances excitatory transmission through a complement-independent mechanism, promoting glutamate ionotropic receptor α-amino-3-hydroxy-5-methyl-4-isoxazolepropionic acid (AMPA) type subunit 1 (GluA1)-S831 phosphorylation and membrane insertion; knockdown of NPTX2 reduces phosphorylated GluA1 and postsynaptic density protein 95 (PSD95) loss, decreases seizure frequency, and reverses cognitive deficits (62). In this acute context, therefore, NPTX2 acts as a pro-excitatory and seizure-promoting molecule independently of its complement-regulatory function. Conversely, in chronic epilepsy patients with cognitive impairment, higher serum NPTX2 levels correlate positively with Mini-Mental State Examination scores and negatively with the EEG slow/fast wave ratio (63), consistent with the complement-inhibitory, synapse-protective role observed in neurodegenerative models. NPTX2 thus appears to exert mechanistically distinct, stage-dependent functions in epilepsy—a complement-independent pro-excitatory effect acutely and a complement-inhibitory protective effect chronically—which together caution against a single directional interpretation of its role and indicate that the therapeutic consequences of targeting NPTX2 may depend critically on disease stage.

##### 3.1.2.2 CSMD1

CSMD1, a synaptic transmembrane protein, has been characterized as an endogenous complement inhibitor. *In vitro* biochemical studies show that CSMD1 binds C1q to limit activation of the classical complement pathway, acts as a cofactor for complement factor I to promote degradation of complement component 4b (C4b) and C3b, and inhibits MAC assembly at the complement component 7 (C7) level, thereby restraining complement-mediated opsonization of synapses (64). Consistent with a complement-regulatory role in the nervous system, CSMD1 deficiency increases the susceptibility of synaptic elements to microglial engulfment in cultured human cortical neurons, and CSMD1-knockout mice exhibit elevated C3 levels, increased C3 co-localization with presynaptic terminals, reduced retinogeniculate synapse numbers, and impaired eye-specific segregation (19). These functional data, however, derive from *in vitro* systems and the developing visual pathway rather than from epilepsy models, and therefore establish CSMD1 as a complement brake without directly demonstrating its involvement in epileptic synapse loss.

A separate line of evidence links CSMD1 to epilepsy through human genetics. Variants in *CSMD1* have been independently associated with developmental and epileptic encephalopathy (DEE) and idiopathic generalized epilepsy (20), and rare variants in the related family members *CSMD2* and *CSMD3*, which are likewise implicated in synaptogenesis, have been associated with epilepsies (65). At present, the genetic association of *CSMD1* with epilepsy and its experimentally defined complement-inhibitory function constitute two distinct evidence streams that have not yet been mechanistically connected; whether loss of CSMD1 complement-regulatory activity directly promotes pathological microglial synaptic pruning in epilepsy remains to be experimentally tested.

##### 3.1.2.3 APOE

ApoE binds C1q and can modulate classical complement pathway activation (16), but the direction of this effect varies with context. In some settings, ApoE forms complexes with activated C1q that dampen unresolvable inflammation (16). In others—the synovial fluid of rheumatoid arthritis patients, for example—ApoE binding to C1q triggers complement activation (66). These opposing outcomes appear to reflect differences in isoform conformation and the local environment. Because most of this work has been done in non-neuronal or neurodegenerative settings, how the same logic applies to synaptic complement regulation in epilepsy is not yet clear.

The clinical genetics tells a separate story. Meta-analyses link the *APOE* ϵ4 allele to increased epilepsy risk, with ϵ2 possibly conferring relative protection (17, 18), and *APOE* genotype has also been implicated in idiopathic generalized epilepsy (67). These associations identify ApoE as a candidate genetic modifier, but the mechanism, particularly the connection to microglial synaptic pruning, has not been worked out. The closest mechanistic clue comes from AD, where reduced binding of ApoE4 to complement factor H promotes amyloid-β (Aβ) oligomerization and neuroinflammation (68), showing that ApoE isoforms can shape complement-dependent processes that affect microglia and synapses. Whether the same applies to complement-mediated pruning in epilepsy, and whether ϵ4 confers its risk through that route, has not been directly tested.

##### 3.1.2.4 SRPX2

SRPX2 is a neuronally expressed complement inhibitor. Its three conserved sushi domains bind C1q and limit classical pathway activation, protecting synapses from complement-mediated elimination reviewed in (69). The strongest functional evidence is developmental: in SRPX2-knockout mice, C3 deposition at synapses increases, microglial engulfment is enhanced, and excitatory synapse density is reduced in the thalamus and cortex, with the synaptic loss shown to be C3-dependent (70). SRPX2 is thus an endogenous brake on complement-driven pruning, but the effect has been documented only for excitatory synapses, and the experiments were done in the context of activity-dependent developmental refinement (70, 71), a setting that does not directly model chronic acquired epilepsy.

The link between SRPX2 and epilepsy comes through separate lines of evidence that have not yet been tied to its complement-inhibitory role. *SRPX2* mutations are associated with Rolandic epilepsy, a pediatric form often accompanied by language and cognitive impairment (72), and *in utero* silencing of Srpx2 in rats produces neuronal migration defects and postnatal epileptiform activity (73). SRPX2 has also been reported to promote excitatory synapse formation independently of complement (74), so its roles in synaptogenesis, neuronal migration, and complement regulation are at least partly separable. What has not been tested is whether loss of SRPX2 complement-inhibitory function specifically drives pathological microglial pruning in the epileptic brain, and whether it acts on inhibitory as well as excitatory synapses.

### 3.2 UTP/UDP

UDP acts on the pruning machinery in a different way from PS or complement components. Where those serve as direct synaptic labels, UDP is an extracellular danger signal that activates microglial phagocytosis through the P2Y6 receptor (P2Y6R). The pathway was originally described in developmental synaptic remodeling, and whether epileptic phagocytosis is essentially a reactivation of the same program is not yet settled (75).

Several lines of evidence indicate that the UDP–P2Y6R axis is engaged during epileptogenesis in KA models. Extracellular uridine triphosphate (UTP)/UDP rises after KA-SE, and P2Y6R expression increases in CA1, CA3, and cortical regions in parallel with microglial activation (75, 76). Because P2Y6R is selectively activated by UDP, receptor signaling presumably depends on extracellular conversion of released UTP to UDP. The strongest epilepsy-specific data link P2Y6R to microglial calcium signaling and lysosome-dependent phagocytosis (75, 77); proposed connections to nuclear factor κB (NF-κB)-dependent inflammatory signaling rest largely on non-epileptic or *in vitro* work and have not been firmly established in epileptic pruning (77).

Functional studies support a pathogenic role for excessive P2Y6R-mediated phagocytosis. Genetic deletion of P2Y6R, pharmacological inhibition with MRS-2578, and blockade of downstream calcium signaling all reduce microglial engulfment, improve CA3 neuronal survival, and preserve cognitive function after KA-SE (75, 78) signaling. Umpierre et al. specifically showed that microglial P2Y6R-dependent calcium signaling is required for phagocytic activity during epileptogenesis, providing a direct link between extracellular nucleotide signaling and microglial engulfment in an epilepsy model (75).

Several gaps still prevent the pathway from being firmly placed in current models of epilepsy-associated pruning. Much of the mechanistic framework for P2Y6R-dependent engulfment comes from developmental or aging studies, and the epilepsy-specific evidence is largely restricted to acute KA models with phagocytosis measured in bulk. The more substantial gap is that the identity of the engulfed synapse type has not been resolved. No study has tested whether P2Y6R-dependent phagocytosis preferentially targets inhibitory (vGAT^+^/gephyrin^+^) or excitatory (vGluT1^+^) synapses, and the distinction matters: more phagocytosis alone does not show whether the pathway contributes to the inhibitory-synapse loss and E/I imbalance that underlie epileptogenesis. Resolving the synaptic specificity of UDP–P2Y6R-mediated engulfment is the central question for judging P2Y6R as an antiepileptic target.

### 3.3 PS

PS exposure at synapses functions as an eat-me signal, mediating synaptic pruning. Physiologically, PS resides mostly in the inner leaflet of the plasma membrane, maintained by ATP-dependent P4-ATPase flippases (79). In the developing retina–lateral geniculate nucleus (LGN) and hippocampal excitatory synapses, only a spatially restricted fraction of PS—primarily presynaptic—is exposed in a caspase-3–independent manner, coinciding with the developmental window of microglial pruning (71, 80). Exposed PS is recognized by microglia through two distinct mechanisms. The direct pathway involves brain-specific angiogenesis inhibitor 1 (BAI1), T-cell immunoglobulin and mucin domain (TIM) family members, TREM2, and G protein-coupled receptor 56 (GPR56), which bind PS through their extracellular domains. The indirect pathway employs bridging molecules: milk fat globule-EGF factor 8 (MFG-E8) links PS to microglial integrin αVβ3 via its discoidin domain, while growth arrest-specific 6 (Gas6) and protein S bridge PS to Tyro3/Axl/Mer (TAM)–family receptors Tyro3, Axl, and Mer tyrosine kinase (MerTK) (79, 81).

In epilepsy, pathological PS externalization at synaptic membranes is driven by two mechanistically distinct routes that converge to amplify microglial phagocytosis beyond developmental levels. First, seizure-induced neuronal stress activates caspase-3 in a non-apoptotic manner, inhibiting flippase activity and permitting irreversible PS exposure through XK-related protein 8 (XKR8) scramblase (82). Second, seizure-associated Ca^2+^ transients activate transmembrane protein 16F (TMEM16F; also known as anoctamin-6), a Ca^2+^-dependent scramblase that catalyzes rapid, reversible PS exposure in viable neurons independently of apoptotic signaling (83). Together, these pathways establish a sustained PS signal at synaptic membranes available for microglial recognition.

Among PS receptors, MerTK occupies a mechanistically privileged position in inhibitory synapse loss. Li et al. demonstrated that neuronal PS exposure—generated by conditional deletion of the flippase chaperone cell division cycle 50A (Cdc50a)—is preferentially displayed on the somatic membrane, the anatomical site of axosomatic GABAergic inhibitory inputs from parvalbumin-positive interneurons. This spatially biased PS presentation directs microglial MerTK-mediated engulfment specifically toward gephyrin-positive inhibitory post-synapses, without affecting excitatory synapses or inhibitory presynaptic terminals. The resulting E/I imbalance is sufficient to generate spontaneous seizures, and both microglial ablation and microglia-specific *Mertk* deletion fully rescue the inhibitory post-synapse loss and the seizure phenotype (80). Critically, this MerTK–PS axis operates in parallel to, and mechanistically independent of, the C1q–C3–CR3 complement pathway, meaning that interventions targeting only complement (e.g., C1q neutralization, C3aR antagonism) would leave MerTK-driven inhibitory synapse loss intact.

Future studies should clarify the spatiotemporal dynamics of PS exposure across the acute, latent, and chronic phases of epilepsy, dissect the relative contributions of XKR8- versus TMEM16F-driven externalization, and test whether selective MerTK inhibitors—currently developed in oncology—provide additive benefit over complement-targeted interventions in epilepsy models.

### 3.4 TREM2

TREM2 plays a dual role within the PS-recognition pathways. It is a PS receptor, but also acts as a brake on complement activation. Under physiological conditions, this combination supports phagocytic capacity while limiting excessive complement-mediated synapse elimination. Loss of TREM2 therefore reduces clearance efficiency, allows PS-exposed synaptic material to accumulate, raises complement activation, and increases network excitability (15, 84–86). There is also evidence that TREM2 recognizes PS on neuronal membranes after excitotoxic injury: TREM2 loss impairs clearance of PS-positive substrates and is associated with spontaneous thalamocortical epileptiform activity, while enhancing TREM2—either with peptides or by neuron-specific overexpression—reduces neuroinflammation, neuronal injury, and seizure burden (15, 87, 88). The net effect in epilepsy so far looks protective. The strongest direct evidence linking TREM2 to PS-driven synapse engulfment still comes from AD, where amyloid-induced PS exposure promotes synaptic phagocytosis (4, 89). Epilepsy-specific studies have mostly documented clearance of damaged neurons and cellular debris in the post-SE period (15), and whether TREM2-dependent phagocytosis preferentially targets inhibitory or excitatory terminals has not been tested.

The two roles, PS recognition and complement restraint, are reasonably well established. The more specific question of whether TREM2 contributes to the selective loss of GABAergic synapses behind chronic E/I imbalance has not been answered. Whether TREM2-dependent phagocytosis stays in the acute debris-clearing mode or shifts toward pathological pruning as disease progresses is still open.

### 3.5 MD2-Clec7a

Whether MD2–CLEC7A signaling acts as an eat-me cue in epilepsy is still an open question; direct evidence is lacking, and what is known comes from other disorders. In ischemic stroke models, neuronal stress induces surface exposure of MD2, which activates the microglial CLEC7A–spleen tyrosine kinase (SYK)–NF-κB/NLRP3 pathway, promotes synaptic engulfment, and contributes to cognitive impairment (21). A similar CLEC7A-dependent pathway operates in tauopathy, where it drives microglia-mediated synapse loss; genetic suppression of CLEC7A or pharmacological antagonism with laminarin preserves synapses and improves memory (22) while. The functional outcome appears to depend on context and intensity: moderate SYK activation may support Aβ clearance in AD models (90), while sustained activation in ischemic and tauopathic settings drives pathological synapse elimination. Extending this to epilepsy is biologically plausible, since seizure-induced excitotoxic stress could in principle expose MD2 at neuronal membranes. The inference is speculative, however. The mechanistic data come entirely from ischemic stroke, tauopathy, and AD models, all of which differ from TLE in injury pattern, inflammatory milieu, and temporal course. For epilepsy, the basic questions are still open: whether MD2 actually reaches the neuronal surface at engaging levels, whether CLEC7A is upregulated in human epileptic tissue, and whether the resulting phagocytosis shows any synapse-type preference. Until these are addressed, the MD2–CLEC7A–SYK axis is best treated as a testable hypothesis for epilepsy, not as an established therapeutic target.

## 4 Don’t-eat-me signals

Don’t-eat-me signals limit how aggressively microglia prune synapses and help prevent the kind of excess phagocytosis that would destabilize the E/I balance. Under physiological conditions, cluster of differentiation 47 (CD47) is enriched at relatively active synapses, and its co-localization with retinal ganglion cell inputs drops in the inhibited eye (91) signaling. CD47–signal regulatory protein α (SIRPα) deficiency in turn reduces synapse numbers in the dorsolateral LGN (dLGN) (71). However, no *in vivo* study has directly tested CD47–SIRPα signaling as a primary interventional variable in epilepsy. The most relevant evidence comes from surgical specimens of refractory epileptic foci in children with FCD type IIb and TSC, where CD47 and SIRPα mRNA and protein are decreased in dysplastic neurons and activated microglia, respectively. CD47 expression correlates inversely with activated microglial density, and recombinant CD47-Fc partially suppresses interleukin-6 (IL-6) release *in vitro* (92). Reduced CD47–SIRPα signaling therefore tracks pathological microglial activation in epileptic tissue, but the available data cannot say whether the change drives activation or merely accompanies it, nor whether restoring the pathway would limit pruning *in vivo*.

Most of what we know about CD47–SIRPα regulation of pruning comes from non-epileptic disorders, and this evidence has to be applied carefully. In AD models, the metabotropic glutamate receptor 5 (mGluR5) modulator BMS-984923 restores synaptic density and reduces C1q-mediated tagging (93), showing that glia-mediated synapse elimination can be modified pharmacologically in amyloid-driven disease, though without acting directly on CD47–SIRPα itself. Progressive reductions in neuronal CD47 or microglial SIRPα in AD and aging models correlate with synapse loss and cognitive impairment (94, 95); whether the same erosion occurs in epileptic circuits has not been shown. In bone cancer pain models, downregulating CD47–SIRPα promotes microglial engulfment of GABAergic synapses in the spinal dorsal horn (96) —anatomically distant from cortical epilepsy, but mechanistically informative. Perioperative neurocognitive disorder models add a further complication: reduced neuronal activity can suppress CD47–SIRPα and enhance engulfment, but single-cell SIRPα overexpression can paradoxically worsen synapse loss (97), suggesting the pathway does not behave linearly. On the available evidence, CD47–SIRPα is a plausible regulator of pathological pruning in epilepsy, but its therapeutic value remains untested—seizure-model studies with cell-type and synapse-type resolved readouts are needed before it can be evaluated as a target.

## 5 Mechanisms of microglia-mediated synaptic pruning in epilepsy

**Figure 3:**
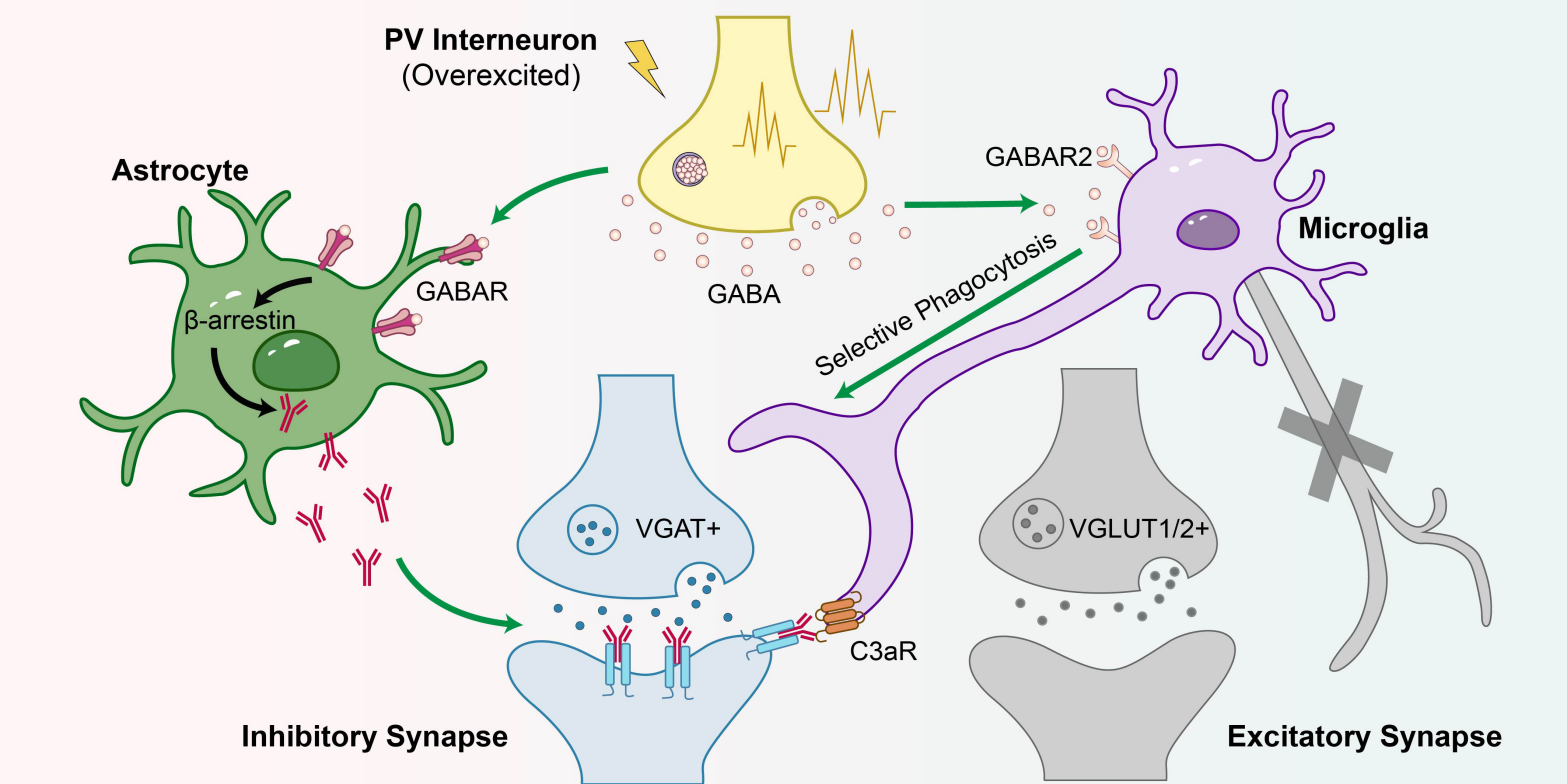
Mechanism underlying selective pruning of inhibitory synapses in epilepsy. Proposed mechanistic model explaining why microglia preferentially eliminate VGAT^+^ inhibitory synapses while sparing VGLUT1/2^+^ excitatory synapses in epilepsy, and how this bias generates a self-reinforcing pro-ictal loop. The cascade is initiated by hyperexcitable parvalbumin (PV) interneurons, which—due to their high firing rates and dense perisomatic innervation—release disproportionately large amounts of GABA into the extracellular space during epileptic activity. This locally elevated GABA acts as the master signal coupling inhibitory hyperactivity to glial recognition through two parallel arms: (i) microglial arm: GABA directly engages microglial GABA_B receptor subunit 2 (GABBR2), polarizing microglia toward a phagocytic state and biasing their surveillance toward GABAergic terminals; (ii) astrocytic arm: GABA activates astrocytic GABA receptors, which recruit β-arrestin and trigger transcriptional/secretory programs that increase astrocyte-derived complement components (notably C3) and complement-tagging activity targeted at VGAT^+^ terminals. The mechanistic basis of selectivity lies at this tagging step: because the GABA signal itself originates from VGAT^+^ terminals, the astrocyte–microglia complement machinery is spatially restricted to inhibitory synapses, leaving adjacent VGLUT1/2^+^ excitatory synapses unmarked and therefore invisible to phagocytic recognition. C3-opsonized VGAT^+^ synapses are then selectively recognized by microglial C3aR, which drives engulfment specifically of inhibitory boutons. The functional consequence is a directional shift in the excitatory/inhibitory (E/I) balance: loss of perisomatic inhibition disinhibits principal neurons, increases PV interneuron drive in a compensatory but ultimately maladaptive manner, releases more GABA, and thereby strengthens the same signaling cascade that triggered selective pruning—closing a feed-forward loop that drives seizure generation, propagation, and chronic epileptogenesis.

During normal development, complement-dependent pruning is not intrinsically biased toward one synapse class. It follows neuronal activity, preferentially eliminating less active glutamatergic or GABAergic contacts (7, 44). In epilepsy, the same molecular machinery can in principle act on both inhibitory and excitatory synapses, but it does not do so symmetrically: the available evidence points clearly toward preferential loss of inhibitory contacts. Chen and colleagues showed that hyperactive parvalbumin interneurons release excessive GABA, which engages both microglial GABA_B_R and a non-canonical astrocytic GABA_B_R–β-arrestin pathway, rapidly inducing C3. The resulting C3 cleavage products, C3b and C3c, are deposited onto gephyrin-positive inhibitory postsynaptic densities. Microglia then accumulate around the somata and proximal dendrites of pyramidal neurons and, through C3aR-dependent recognition, engulf these tagged inhibitory terminals (11). Three-dimensional electron microscopy and high-resolution confocal imaging further showed an approximately 40% reduction in vGAT^+^/gephyrin^+^ inhibitory synapse density in the CA1 stratum radiatum, while vGluT1^+^ excitatory terminals were not co-engulfed and may even accumulate in parallel (10, 11). Consistent with this, microglia preferentially internalize GAD65^+^/vGAT^+^ inhibitory terminals, while vGluT1^+^ excitatory terminals are relatively, or even absolutely, preserved (10, 11). Functionally, this selective inhibitory synapse loss is associated with an approximately 50% reduction in the frequency of spontaneous inhibitory postsynaptic currents (sIPSCs), an increased E/I current ratio, enhanced gamma-band synchrony, and a lower seizure threshold (11, 98). The proposed mechanism is summarized in **Figure 3**. Interventions targeting the GABA_B_R–C3–C3aR axis prevent inhibitory synapse loss, restore E/I balance, reduce seizures by approximately 60–70%, and attenuate cortico-hippocampal gamma synchrony (11, 27). As discussed in Section 3.3, the MerTK-mediated complement-independent pathway may contribute to the same outcome and is not revisited here.

Although microglia in TLE display a clear preference for engulfing inhibitory synapses (10, 11), pruning of excitatory synapses is not absent from the epileptic brain; rather, it differs fundamentally in timing, context, and pathological significance. *In vivo*, excitatory synapse pruning is observed mainly during the acute to subacute period after seizures and appears to reflect either passive clearance of structurally damaged synapses following excitotoxic injury or a compensatory form of homeostatic plasticity. Seven days after electrically induced SE, for example, there is a transient drop in PSD95-positive puncta on the dendrites of newborn dentate granule cells, consistent with downscaling in response to excessive excitation (99). Microglial accumulation of vGluT1/PSD95 signals has also been reported in specific genetic models, such as activated C kinase 1 (RACK1)-mutant mice, and in viral encephalitis-associated epilepsy (2, 100). Once chronic SRS are established, however, any continuing pruning of excitatory synapses tends to be masked by aberrant excitatory synaptogenesis—recurrent mossy fiber sprouting into the inner molecular layer of the dentate gyrus being the clearest example. Total vGluT1 immunoreactivity across multiple hippocampal subregions remains elevated, and there is little to no colocalization between vGluT1 and cluster of differentiation 68 (CD68)-positive microglial lysosomes (10, 101). Excitatory synapse pruning in epilepsy therefore looks like a transient, acute-phase phenomenon, in contrast to the sustained engulfment that drives inhibitory synapse loss.

## 6 Therapeutic strategies targeting microglial synaptic pruning in epilepsy

Designing pruning-targeted therapy in epilepsy comes down to three connected questions: when in the disease course to intervene, how to redirect microglial state, and which molecular checkpoints in the pruning cascade are specific enough to target without collateral damage. The subsections below take these up in turn—disease stage (Section 6.1), activation and polarization (Section 6.2), and the find-me, eat-me, and don’t-eat-me recognition pathways (Section 6.3). Pruning is not equivalent to inflammation, and a generic anti-inflammatory approach is unlikely to address it. Because microglial behavior changes substantially across epileptogenesis, the same compound can be protective at one stage and harmful at another. **Table 1** summarizes the main preclinical and clinical agents discussed below.

**Table 1:** Molecular targets of microglial pathological synaptic pruning in epilepsy.

Agent	Target	Model	Effect on pruning/microglia (preclinical)	Anti-seizure outcome (preclinical)	Clinical evidence/status	Limitations	References
Anti-C1q neutralizing antibody	C1q	Mouse, pilocarpine	Reduced complement-mediated synaptic opsonization; attenuated microglial phagocytic transition	Reduced interictal spike frequency over 30-day window	None; no clinical epilepsy data	Single model; post-SE administration only; no inhibitory vs. excitatory synapse quantification	(56)
C1 esterase inhibitor (C1-INH)	Classical pathway (upstream)	Rat, pilocarpine	Paradoxically increased synaptic loss and microglial activation	No improvement in cognitive deficits	None in epilepsy (approved for hereditary angioedema)	Upstream node suppression; paradoxical outcome illustrates node-specificity	(133)
Captopril	ACE → astrocytic C3↓ → C3aR	Rat, KA	Reduced astrocytic C3 and microglial C3aR; attenuated synaptic phagocytosis in CA subregions	Suppressed SRS; improved Morris water maze and Y-maze	Approved antihypertensive; not tested for pruning in epilepsy	Inhibitory vs. excitatory ratio not quantified; ACE inhibition affects pathways beyond C3	(129)
Cobra venom factor (CVF)	C3 (global depletion)	Mouse, pilocarpine	Global C3 depletion	Alleviated SE-induced cognitive impairment	None; not clinically translatable	Non-selective; not translatable	(134)
Salidroside	C3–C3aR axis	Rat, PTZ kindling	Downregulated C3 and C3aR in microglia and astrocytes; reduced cytokine release	Prolonged seizure latency; reduced severity and cognitive deficits	None in epilepsy	Broad anti-inflammatory; no synapse-type–specific endpoint	(130)
H_2_S donor compounds	C3–C3aR axis	Mouse/rat, pilocarpine	Suppressed C3–C3aR signaling; reduced microglial activation markers	Reduced interictal spike frequency and severity	None in epilepsy	Mechanism not restricted to pruning; no inhibitory synapse density data	(131, 132)
C5aR1 antagonist (PMX53)	C5a–C5aR1	Animal, epilepsy-relevant injury	Reduced C5aR1-driven microglial activation	Attenuated injury/hyperexcitability	None in epilepsy	Limited epilepsy-specific data; pruning endpoint not measured	(107)
Anti-CX3CR1 antibody	CX3CR1	Rat, electrical kindling	Suppressed microglial overactivation and pathological chemoattraction	Reduced microglial-associated neuronal injury	None	No SRS frequency endpoint; seizure outcome data lacking	(39)
JNJ-54175446	P2X7R	Mouse, KA (drug-resistant TLE)	Suppressed P2X7R–NLRP3–IL-1β–driven activation	Reduced seizure frequency in drug-resistant TLE model	No epilepsy data (P2X7R antagonists in trials for other CNS indications)	Drug-resistant model only; pruning-specific outcome not measured	(124)
JNJ-47965567	P2X7R	Mouse/rat, pilocarpine	Promoted anti-inflammatory microglial polarization	Reduced seizure severity and duration in chronic TLE model	None (preclinical tool compound)	No direct synapse-type quantification	(125)
JNJ-42253432	P2X7R	Rat, KA	Not reported	Reduced severe seizures; increased proportion of mild seizures	None (preclinical tool compound)	No pruning or microglial mechanistic endpoint reported	(125)
P2X7R siRNA	P2X7R	Rat, pilocarpine	Reduced P2X7R-mediated microglial activation	Prolonged SRS latency; reduced SRS frequency and severity	None	siRNA delivery limitations; pruning-specific mechanism unconfirmed	(126)
Brilliant Blue G (BBG)	P2X7R	Rat, Li-Pilo	Suppressed microglial activation	Attenuated depression- and anxiety-like comorbid behaviors	None	Behavioral outcome only; no seizure frequency or synapse-type endpoint	(123)
Astaxanthin (AST)	P2X7R	Rat, Li-Pilo	Suppressed P2X7R–NLRP3–IL-1β–driven activation	Alleviated SE-induced cognitive impairment	Dietary compound; no epilepsy clinical trial	Cognitive endpoint only; no seizure frequency or synapse-type data	(122)
JNJ-47965567 + KW6002/SCH58261	P2X7R + A2AR	Mouse/rat, KA	Not reported	Prevented cognitive impairment; reduced severity; altered SE latency/duration	None	Combination; individual contributions not dissected; no synaptic endpoint	(128)
MRS-2578	P2Y6R	Rat/in vitro, KA	Suppressed UDP–P2Y6R–driven lysosomal biogenesis and excessive phagocytosis	Improved CA3 neuron survival; cognitive benefit	None (preclinical tool compound)	Most evidence in vitro; in vivo seizure frequency endpoint not reported	(75, 78)
MerTK blockade	PS–MerTK	Mouse, post-SE	Interrupted PS-bridging engulfment; rescued inhibitory synaptic architecture	Improved epileptic phenotype	None	Specification largely developmental/non-epileptic	(80)
TREM2 augmentation (peptide/overexpression)	TREM2	Mouse, acute & chronic	Enhanced clearance; restrained complement	Reduced seizure frequency	None	Dual role; loss is harmful; enhancement (not inhibition)	(87, 88)
Laminarin	MD2–CLEC7A	Ischemia, tauopathy (no epilepsy)	Blocked CLEC7A–SYK phagocytic signaling; rescued synapses	Not tested in epilepsy	None	No direct epilepsy evidence	(21, 22)
MEF2A inhibition (AAV-shMEF2A; parecoxib)	MEF2A	KA; human TLE tissue	Reduced microglial engulfment of GABA_a_R/inhibitory synapses	Reduced seizures	None (parecoxib approved as analgesic, not for this use)	Single-pathway finding; needs independent replication	(36)
CD47-Fc/mGluR5 SAM	CD47–SIRPα	Pediatric resected tissue; in vitro; AD	Reinforced don’t-eat-me checkpoint; restored CD47 (mGluR5 SAM, AD)	Not directly tested in epilepsy	None in epilepsy	No in vivo epilepsy intervention; mGluR5 data extrapolated from AD	(92, 93)
IVIG	Multi-node immune modulation	Selected clinical epilepsies	Not synapse-resolved	Clinical benefit in selected epilepsies	Clinical use in selected epilepsies (e.g., autoimmune/refractory)	Inhibitory-synapse rescue not measured; mechanism non-specific	(135)
Minocycline	Broad microglial inhibition	Multiple rodent models; limited human case series	Non-selective inhibition of microglial activation and pro-inflammatory polarization	Variable anti-seizure effects across models	Limited observational/case-series data in epilepsy; non-specific	Non-selective; risk of suppressing protective acute-phase microglia; no synapse-type endpoint	(112)
Metformin	Microglial activation (metabolic)	Pilocarpine	↓ microglial activation and CD11b^+^ density	↓ seizure frequency and mortality	Widely approved (diabetes); no epilepsy pruning trial	Metabolic pleiotropy; no synapse-type readout	(113–115)
Dapagliflozin	Microglial activation (metabolic)	KA-SE	↓ microglial CD68 and iNOS	Reduced seizure severity	Approved (diabetes); no epilepsy data	Indirect; acute-phase only	(116)
AM1241	CB2 receptor	KA	Pro-inflammatory → reparative microglial polarization	Improved seizure and behavioral outcomes	None	Phenotype-marker based; not pruning-resolved	(117)
Plant-derived compounds	Microglial activation/inflammation	Various	Microglial deactivation; anti-inflammatory polarization	Reduced seizures across models	None	Mechanistically non-specific; no synapse-type endpoint	(118–121)

SRS, spontaneous recurrent seizures; KA, kainic acid; Li-Pilo, lithium–pilocarpine; PTZ, pentylenetetrazol; TLE, temporal lobe epilepsy; SE, status epilepticus; ACE, angiotensin-converting enzyme; CVF, cobra venom factor; BBG, Brilliant Blue G; AST, astaxanthin; IVIG, intravenous immunoglobulin; SAM, subtype-selective allosteric modulator. All agents are preclinical unless otherwise stated in the clinical column. No agent has been evaluated in a randomized controlled trial with synaptic pruning as a primary endpoint.

### 6.1 Stage-dependent microglial behavior: rationale for temporally gated intervention

Microglia do not contribute to epilepsy in a single way. Their role changes substantially across the acute insult, the latent period, and the chronic state, and the direction of change matters for therapy: the same population that helps stabilize circuits in the first hours after seizure can drive synapse loss weeks later. Any pruning-targeted strategy therefore has to start from this temporal picture. Ignoring it risks dampening a useful early response, or missing the window when intervention would actually help.

During the first 1–3 days after the precipitating insult, microglial responses look mainly protective. Homeostatic microglia continuously monitor neighboring neurons through P2Y12R-dependent somatic purinergic junctions and provide negative feedback on hyperactive cells (24, 27). This surveillance continues during the acute epileptic response and seems to be co-opted into it: in *P2ry12*-deficient mice, the acute microglial morphology change is lost, KA-induced seizures become more severe, and seizure-associated neuronal loss worsens (102, 103). Microglial preconditioning paradigms point the same way—low-dose lipopolysaccharide given before epileptogenesis activates microglia in a state associated with reduced glutamatergic spine density and neuroprotection (98, 104). When microglia are removed during this window, with conditional depletion or colony-stimulating factor 1 receptor (CSF1R) inhibition by PLX5622 in the first 24–72 hours after SE, acute seizures worsen and hippocampal degeneration accelerates in both pilocarpine and KA models (105, 106). Indiscriminate suppression in the peri-ictal period therefore looks risky. At this point in the disease, broad inhibition removes a useful buffering response without actually engaging an established pruning program.

The latent phase, extending from several days to approximately two weeks after injury, is more therapeutically ambiguous but arguably more important for disease modification. This period is characterized not simply by persistent inflammation, but by the progressive reorganization of glial signaling networks before SRS become established. Microglial polarization shifts dynamically over this interval, varying with model, region, and time (107, 108). Several lines of evidence place microglia upstream of subsequent astrocytic changes: in drug-induced SE models, microglial M2 polarization precedes astrocytic reactivity and is required for the later development of astrocytic morphological changes and inositol 1,4,5-trisphosphate receptor type 2 (IP3R2)-dependent Ca^2+^ hyperactivity (109). Hippocampal CD68 expression also rises persistently between days 7 and 14 after KA-SE, in parallel with dendritic spine remodeling (110), consistent with microglia acquiring a more phagocytic phenotype during this window. Timing within the latent phase matters as well. PLX5622-mediated microglial depletion during the first week after SE suppresses later astrocytic reactivity and reduces subsequent seizure susceptibility, while the same intervention started three weeks after SE no longer prevents astrogliosis (109). That distinction is relevant for the astrocytic GABA_B_R–β-arrestin–C3 branch of the inhibitory-pruning pathway, which depends on astrocytic activation (11). Practically, this makes the latent phase an upstream point of intervention—a window in which maladaptive glia–glia signaling can still be interrupted before chronic synapse loss is locked in.

By the chronic phase, extending from weeks to months after the initial insult, the case for pruning-targeted intervention becomes mechanistically stronger. In chronic TLE, hippocampal microglia adopt a persistent complement-enriched, phagocytically active phenotype, characterized by sustained upregulation of C1q, C3, CD68, and lysosomal genes (10, 13, 55). Transcriptomic analyses of human and experimental hippocampus identify complement activation and phagocytosis as defining features of this stage (111). It is within this context that pathological pruning is most clearly expressed as a disease-sustaining process rather than an acute response to injury. The GABA–astrocyte–C3–C3aR axis selectively removes inhibitory synapses (11), and MerTK-dependent recognition of PS-exposed inhibitory postsynaptic structures drives further loss of inhibition (80). Interventions aimed at the downstream effectors of this cascade have shown functional benefit during this phase: complement antagonism with C3aR blockade or anti-C1q antibodies reduces seizure frequency and partially restores E/I balance (56). The chronic stage thus seems to be the most plausible therapeutic window for direct interference with pruning. The challenge here is to suppress maladaptive engulfment without losing the homeostatic functions microglia still perform in mature tissue.

Across these phases, when to intervene matters at least as much as what to target. Acute microglia are largely doing useful work and should not be globally suppressed. Latent-phase microglia are a more strategic point of intervention, because their behavior during this window helps determine whether astrocytic reactivity and phagocytic competence develop downstream. Chronic microglia, by contrast, are the population most directly tied to the complement-rich engulfment programs that drive inhibitory synapse loss. One practical consequence is that the same drug can have very different effects depending on when it is given. A compound effective in chronic epilepsy may be useless or actively harmful during the acute injury response, while a latent-phase intervention may help most by preventing the emergence of pruning-permissive circuitry rather than by reversing it later.

### 6.2 Pharmacological modulation of microglial activation and phenotypic polarization

Given how strongly microglial behavior depends on disease stage, “suppressing microglia” is no longer a satisfactory framing for pharmacological intervention. A more useful goal is to redirect microglial state—keeping the surveillance and debris-clearing functions that matter in the acute post-insult period, while limiting the chronic, pro-inflammatory, engulfment-prone programs that drive maladaptive synapse loss. The depletion paradigms discussed in Section 6.1 illustrate the risk of treating this too crudely: blunting microglial activity during the acute window can remove a protective response and worsen outcome, even when similar manipulations might help in chronic disease. The real question now is which microglial states should be targeted, and at what stage.

A range of agents with broad microglia-modulating activity have shown some efficacy in preclinical models. Minocycline, a tetracycline long known for its microglia-suppressive effects, attenuates seizures in several rodent models and has been tried in a few clinical settings, but its mechanism is non-specific, and the anti-seizure effect cannot be cleanly attributed to reduced pruning rather than to anti-inflammatory action more generally (112). Metabolic drugs have attracted interest on similar grounds. In pilocarpine models, metformin reduces microglial activation and cluster of differentiation 11b (CD11b)^+^ cell density in the hippocampus, thalamus, and piriform cortex, while also lowering seizure frequency and mortality (113–115). Dapagliflozin given after SE downregulates microglial CD68 and inducible nitric oxide synthase (iNOS) expression (116). The cannabinoid receptor type 2 (CB2) agonist AM1241 likewise appears to bias microglia toward a reparative phenotype, reducing ionized calcium-binding adapter molecule 1 (Iba1)^+^/cluster of differentiation 16 (CD16)^+^ pro-inflammatory cells and IL-1β while increasing Iba1^+^/arginase 1 (Arg1)^+^ cells, with accompanying improvements in seizure and behavioral outcomes (117). Several plant-derived compounds and astaxanthin produce broadly similar profiles of microglial deactivation and anti-inflammatory polarization across epilepsy models (118–122).

None of these compounds has been shown to act directly on the pruning machinery. The endpoints in these studies tend to be cytokine levels, generic microglial activation markers, or seizure counts, rather than synapse-resolved measures such as vGAT^+^/gephyrin^+^ inhibitory bouton density. The reductions in seizures they produce may therefore reflect broad anti-inflammatory or neuroprotective effects rather than a targeted reduction in pathological engulfment. That matters here: if the rationale for the intervention is to prevent the preferential loss of inhibitory synapses, the relevant outcome is preservation of those terminals, not a drop in inflammatory markers. The current literature does not yet support calling phenotype-biasing agents anti-pruning drugs in any mechanistically specific sense.

Two other issues compound this. Most supportive data come from acute KA or pilocarpine models, where reductions in seizures may mainly reflect mitigation of acute inflammatory injury rather than correction of chronic pruning pathology. And the timing problem returns here too: an agent that helps in the chronic phase could be harmful if given during the acute window, when microglia are still doing protective work. On balance, phenotype-biasing approaches are mechanistically plausible and reproducibly active preclinically, but whether they specifically affect pathological pruning, when they should be deployed, and how well they cross the blood–brain barrier into chronic human TLE all remain unsettled.

### 6.3 Targeting find-me, eat-me, and don’t-eat-me signaling pathways

An alternative to broad microglial modulation is to act directly on the molecular recognition system that decides whether a synapse is engulfed. Find-me, eat-me, and don’t-eat-me checkpoints offer specific molecular handles, and the candidate targets and corresponding agents are summarized in **Tables 2** and **1**. The difficulty is that the evidence behind individual nodes is very uneven. Some pathways have been directly validated in epilepsy models, while others are promoted largely on the strength of work in development, Alzheimer’s disease, or stroke, and this distinction matters when prioritizing what to take forward.

**Table 2:** Molecular targets of microglial pathological synaptic pruning in epilepsy.

Signal category	Molecule/target	Key receptor/ligand		Effect on synapses	Epilepsy-specific evidence	References
Find-Me	ATP (low)	P2Y12R		Homeostatic surveillance; protective microglial recruitment	Direct: P2Y12R markedly downregulated in human drug-resistant TLE hippocampus	(23)
	ATP (high)	P2X7R → NLRP3–IL-1β		Pathological activation; drives excessive phagocytosis	Direct: P2X7R upregulated in human MTLE; antiseizure effects of antagonists in multiple rodent models	(32–35)
	CX3CL1	CX3CR1		Pathological chemoattraction; supraphysiological synaptic engulfment	Indirect: CX3CR1^+^ microglial overactivation in rodent seizure models	(37, 38)
	GABA	GABBR2 (microglial)		Selectively recruits microglia to GABAergic terminals; drives inhibitory-specific pruning	Direct: validated in KA mouse model and human TLE surgical tissue	(11)
Eat-Me	C1q	C3 → CR3 (downstream)		Opsonizes weak or damaged synapses for microglial engulfment	Direct: C1q elevated in human refractory TLE tissue; co-localizes with microglia and dendrites; caspase-3–dependent C1q tagging after febrile seizures	(12–14)
	C3/iC3b	CR3 (microglial)		Promotes phagocytosis; inhibitory synapse preference in TLE	Direct: elevated iC3b in human TLE; C3b/C3c deposition at gephyrin-positive inhibitory synapses; C3-dependent pruning in mTBI-acquired epilepsy	(13, 14, 129)
	C5a	C5aR1 (microglial)		Amplifies complement-driven microglial activation and phagocytic signaling	Indirect: C5aR1 antagonism (PMX53) attenuates epilepsy-relevant injury in animal models	(107)
	UDP	P2Y6R		Extracellular danger signal; amplifies the phagocytic program via lysosomal biogenesis	Direct: P2Y6R mRNA upregulated in CA1/CA3 post-KA-SE; in vivo and in vitro phagocytosis evidence	(75, 78)
	Phosphatidylserine (PS)		MerTK, TIM family, BAI1, TREM2, GPR56	Eat-me tag on inhibitory postsynaptic membranes; mediates PS-dependent engulfment	behaviorDirect: MerTK blockade rescues inhibitory synaptic architecture and epileptic phenotype in mouse hippocampus	(80)
	TREM2		PS, ApoE, C1q	Clears PS-exposed synapses; functions as complement brake via C1q capture	Indirect (AD models); limited direct evidence from post-SE clearance studies; no synapse-type–specific pruning data in acquired epilepsy	(4, 15)
	MD2–CLEC7A		CLEC7A–SYK–NF-κB/NLRP3	Drives excessive synapse loss; dose- and context-dependent	No epilepsy functional evidence; all mechanistic data extrapolated from ischemia and tauopathy models	(21, 22)
Complement Inhibitors	CSMD1		C1q (binding inhibition); cofactor for complement factor I	Protects synapses from C1q/C3-mediated attack	No direct epilepsy functional evidence; genetic association with DEE and IGE only	(19, 20, 64)
	ApoE (ϵ2/ϵ3)		C1q (binding inhibition)	Inhibits classical complement pathway activation	No direct epilepsy functional evidence; epidemiological association between APOE ϵ4 and increased epilepsy risk	(16, 18, 66)
	SRPX2		C1q (binding inhibition)	Protects synapses in thalamus and cortex from complement-mediated elimination	Indirect: SRPX2 mutations associated with Rolandic epilepsy; in utero silencing causes epileptiform activity in rodents	(70, 72, 73)
	NPTX2		C1q (co-localization and modulation)	Stage-dependent: pro-excitatory and seizure-promoting in acute phase; neuroprotective in chronic phase	Indirect: opposing effects documented across acute and chronic epilepsy model studies and patient serum data	(61, 63)
Don’t-eat-me	CD47		SIRPα (microglial)	Prevents microglial phagocytosis of active synapses	Indirect: CD47 and SIRPα downregulated in resected FCD IIb and TSC epileptic tissue; CD47 expression inversely correlates with activated microglial density	(91, 92)

TLE, temporal lobe epilepsy; MTLE, mesial TLE; KA, kainic acid; SE, status epilepticus; mTBI, mild traumatic brain injury; DEE, developmental epileptic encephalopathy; IGE, idiopathic generalized epilepsy; FCD, focal cortical dysplasia; TSC, tuberous sclerosis complex; AD, Alzheimer’s disease. “Direct” denotes evidence obtained in epilepsy models or human epileptic tissue; “indirect” denotes evidence extrapolated from related disorders or developmental systems.

Among find-me signals, purinergic receptors have produced the most tractable therapeutic candidates. P2X7R is the most developed: antagonists such as Brilliant Blue G and several structurally distinct compounds reduce NLRP3–IL-1β signaling and seizure burden in rodent models (123–128). These results support P2X7R as a reasonable anti-inflammatory and anti-seizure target, although they do not yet show that the benefit comes specifically from less pathological pruning rather than from a general reduction in inflammation. CX3CR1 blockade attenuates the chemoattractant loop in seizure models (39), but again the readouts are mostly inflammatory rather than synapse-selective. P2Y12R sits in the same broad receptor family and yet points in the opposite direction. It supports homeostatic surveillance and is already downregulated in human TLE, so antiplatelet P2Y12R inhibitors such as clopidogrel and ticagrelor are likely to be counterproductive in this setting (23). Thus, even within a shared signaling class, therapeutic direction depends on physiological function rather than receptor family membership.

The strongest case for mechanism-based intervention currently lies in the eat-me axis, especially within the complement cascade. Complement is the best-characterized synapse-tagging pathway in epilepsy, and it also illustrates most clearly why node selection matters. Downstream interventions have generally helped. Anti-C1q neutralizing antibodies reduce complement tagging and excessive engulfment (56). Captopril, which modulates C3, reduces microglial activation and synapse loss while improving seizures and cognition (129). C3aR antagonism, achieved with salidroside or H_2_S donors, prolongs seizure latency and reduces severity (130–132). The C5a–C5aR1 branch is less developed but has been targeted in epilepsy-relevant injury models (107). The most informative negative data, however, lie upstream: C1 esterase inhibitor (C1-INH) paradoxically increased synapse loss and microglial activation without improving cognition (133), and cobra venom factor-mediated complement depletion likewise suggests that indiscriminate upstream interference can be ineffective or harmful (134). Treating complement as a single deleterious cascade to be shut down therefore misses an important point. Selectively blocking the downstream effectors that drive pathological tagging seems more promising than interfering with upstream initiation, which the system also needs for host defense and routine tissue surveillance. Intravenous immunoglobulin (IVIG), already used in selected epilepsies, acts as a multi-node immunomodulator consistent with this logic, though whether it actually rescues vulnerable inhibitory synapses has not been directly examined (135–141).

Outside the complement system, the UDP–P2Y6R axis is notable because it is one of the few pathways for which the chain from receptor activation, through lysosomal phagocytosis, to functional rescue has actually been pieced together in an epilepsy model. P2Y6R deletion, blockade of downstream calcium signaling, and the selective antagonist MRS-2578 all reduce excess phagocytosis and improve neuronal survival and cognition after KA-induced status epilepticus (75, 78). That makes it among the better-validated phagocytic checkpoints in this field. The remaining gap is that we still do not know what kind of synapse is being spared. Whether P2Y6R inhibition preserves the inhibitory terminals that matter most for the E/I balance has not been shown directly, and until it is, the pathway is best described as promising rather than mechanistically closed.

The PS–MerTK/Axl axis is another node central to phagocytic recognition. MerTK blockade restores synaptic architecture and improves epileptic phenotypes in mice (80), but much of the detailed mechanistic framework still derives from developmental or non-epileptic systems. TREM2 complicates the picture further in a conceptually important way. Because it functions both as a PS receptor and as a brake on complement-driven engulfment. Losing TREM2 is harmful; augmenting it, through peptides or neuronal overexpression, reduces seizure frequency (87, 88). TREM2 therefore represents a candidate for enhancement rather than inhibition, underscoring the broader point that not all phagocytic receptors are pathological drivers; some may instead restrain maladaptive pruning. Several other candidates are at earlier stages. MD2–CLEC7A antagonism with laminarin preserves synapses in ischemia and tauopathy, but no comparable epilepsy study has been done (21, 22). Similarly, the seizure-induced ATP–P2X7R–CaMKII–HDAC5–MEF2A cascade converging on microglial GABA_A_R phagocytosis points to an intriguing transcriptional control node, and both adeno-associated virus (AAV)-shMEF2A and the MEF2A inhibitor parecoxib have been reported to reduce GABA_A_R engulfment and seizures (36). This is an interesting transcriptional control point, but it currently rests on a single study and needs independent replication.

The don’t-eat-me arm is appealing in principle but thinner experimentally. Reinforcing checkpoints like CD47–SIRPα could in theory protect vulnerable synapses without dampening microglia overall. The epilepsy data, though, are still sparse. CD47–SIRPα is downregulated in resected pediatric epileptic tissue, where its decline tracks microglial activation, and recombinant CD47-Fc suppresses IL-6 release *in vitro* (92), but no *in vivo* epilepsy study has yet shown that restoring CD47 rescues synapses or alters seizure outcomes. In other models the pathway can even behave non-linearly at single-cell resolution. Allosteric modulation of mGluR5 restores neuronal CD47 and reduces C1q-mediated tagging in AD (93), suggesting an indirect route into don’t-eat-me signaling, but this has not been tested in seizure models. Much the same caution applies to endogenous complement inhibitors—NPTX2 (61, 63), ApoE (16–18), SRPX2 (69, 70), complement factor H (CFH), and CSMD1 (19, 20, 64). These proteins are useful as biological templates for how C1q–C3 tagging can be restrained, but none has been shown to regulate pruning in adult epileptic tissue, and for SRPX2 and CSMD1 in particular the existing data come mainly from development or genetic association.

Taking the field as a whole, the list of plausible targets is much longer than the list of well-validated ones. The strongest epilepsy-based support currently sits with the downstream complement effectors (C1q, C3, C3aR) and with UDP–P2Y6R. Most other pathways are mechanistically attractive but rest largely on inference from related diseases or developmental work. A few practical caveats recur throughout this literature. Many interventions are scored by total phagocytosis or by inflammatory markers, without confirming that the synapses being spared are actually the vGAT^+^/gephyrin^+^ inhibitory terminals relevant to E/I imbalance. Most preclinical work also still relies on acute KA or pilocarpine models, which do not capture chronic human TLE well; and because early microglia can be protective, the timing of an intervention can determine whether it helps or hurts. There is also a more fundamental issue: complement and phagocytic recognition do not exist solely to drive disease—they are part of host defense and routine synaptic maintenance, so untargeted manipulation risks trading one circuit problem for another. Add to this the usual translational obstacles, including BBB penetration and the lack of tools precise enough to act on the right cell type, synapse type, and disease stage, and the realistic goal becomes clearer. What looks most likely to work is a stage-aware, node-specific intervention that disrupts maladaptive pruning while leaving normal immune surveillance and synaptic turnover largely intact.

## 7 Conclusions and future directions

Microglia-mediated synaptic pruning has emerged as an important non-neuronal mechanism in epileptic circuit remodeling. Beyond their role as reactive immune cells, microglia actively shape synaptic architecture by reading find-me, eat-me, and don’t-eat-me cues from neurons and astrocytes. When this readout is disturbed by chronic seizures, pruning becomes maladaptive: inhibitory contacts are preferentially lost, the E/I balance shifts, and circuits become more excitable and less stable. The recent identification of GABA-driven astrocyte–microglia signaling and complement-dependent removal of inhibitory synapses has been particularly instructive, giving the field a more concrete mechanistic picture than was available even a few years ago. Much of the picture, however, is still incomplete. Complement is the pathway we understand best, but it is only one piece of the recognition machinery. Many find-me signals, several non-complement eat-me ligands, and most don’t-eat-me checkpoints have been studied far more thoroughly in development, Alzheimer’s disease, or stroke than in epilepsy itself. For pathways such as ATP–P2Y12/P2X7R, CX3CL1–CX3CR1, UDP–P2Y6R, PS receptors, and CD47–SIRPα, the epilepsy-specific data are uneven, and conclusions often rely on extrapolation from other disorders. The molecular map of pathological pruning in epilepsy is therefore better described as work in progress than as a finished signaling framework.

Why pruning in epilepsy hits inhibitory synapses harder than excitatory ones is still an open question. Recent studies make a strong case that this selectivity exists, but they do not yet explain it. We do not know why some PV-interneuron boutons are tagged while neighboring inhibitory contacts survive, nor how microglia weigh competing inputs—neuronal activity, astrocytic C3, PS exposure, local metabolism—when deciding which terminal to engulf. Until the rules of selectivity are clearer, it will be hard to know whether a given intervention actually rescues the inhibitory synapses that matter, or only damps down general inflammation.

Microglia themselves are also not a uniform entity. Their behavior changes from the acute insult, through the latent period, to the chronic epileptic state, and probably differs across brain regions and seizure models as well. Acutely, they appear to limit excitotoxic damage and clear debris. During the latent phase, they help shape the astrocytic and phagocytic environment that later supports chronic dysfunction. By the chronic stage, they have settled into a complement-rich, engulfment-prone phenotype that reinforces network instability. The familiar M1/M2 dichotomy does not capture this trajectory well; it collapses what is really a spectrum into two boxes. Stage-resolved and single-cell descriptions will be needed if microglial states are to be linked meaningfully to pruning biology.

These uncertainties have practical consequences for drug development. Many preclinical interventions—against complement components, C3aR, CX3CR1, P2X7R, and microglial activation more broadly—reduce synapse loss and improve seizure outcomes in rodent models. Whether they do so by blocking pathological pruning specifically, or simply by lowering overall neuroinflammation, is rarely tested. The two possibilities have different implications for translation, and so far the field has tended to infer pruning rescue from seizure data rather than measure it directly at the level of vulnerable inhibitory terminals.

Two further complications make translation harder. Microglia are not uniformly harmful: acute microglial responses help clear damaged tissue and may even constrain hyperexcitability, so blunt suppression can backfire, as several depletion studies in acute models have shown. And pathway specificity matters within the cascade itself—downstream complement inhibition tends to help, while broad upstream interference has sometimes worsened outcomes, with C1-INH being the clearest example. On top of this, current tools rarely distinguish among cell types, synapse types, disease stages, or brain regions with enough precision, so an intervention aimed at pathological pruning may also disturb physiological remodeling and normal plasticity.

Several directions therefore seem worth pursuing. The most basic gap is a finer-grained map of how find-me, eat-me, and don’t-eat-me signals interact in epileptic tissue rather than in development or Alzheimer’s models. This includes asking which checkpoints are genuinely causal rather than merely correlated with seizure activity, and which are accessible to pharmacology. Synaptic vulnerability itself deserves more resolution: it remains unknown whether the same selectivity rules apply across hippocampal subregions, seizure etiologies, interneuron subtypes, or pre- versus postsynaptic compartments, and these distinctions may matter clinically. Astrocytes deserve a larger role in this picture than they currently have. The GABA–astrocyte–C3 axis showed that complement induction in epilepsy can be driven by astrocytes rather than by microglia themselves, and similar glia–glia handoffs probably exist elsewhere in the cascade. Animal work also needs more sustained translation into human tissue via spatial transcriptomics, single-cell sequencing, and high-resolution imaging of resected epileptic specimens, so that we can tell whether the pruning signatures defined in rodents are the same ones operating in patients. Therapeutically, interventions that act on a single node, at a defined disease stage, and with a measurable effect at the synaptic substrate are more likely to disturb pathology without unwinding the homeostatic functions microglia continue to perform.

The open question is no longer whether microglia contribute to epilepsy. It is how, when, and at which synapses they cross from useful to harmful, and which parts of that transition can be reversed without losing what microglia normally do well. If those questions can be answered, treatments targeting pruning may eventually offer something current antiseizure drugs cannot, namely the preservation of inhibitory circuitry and some protection against the cognitive decline that so often accompanies chronic epilepsy.
